# RNA biomarker signatures for prediction of acute kidney injury in acute coronary syndrome patients undergoing PCI

**DOI:** 10.1186/s12882-026-04926-w

**Published:** 2026-04-14

**Authors:** Radwa Khaled, Emad El-Zayat, Mohamed A. Ragheb, Sara Elsayed Abdelrahman, Marwa Matboli

**Affiliations:** 1 Biotechnology Department, Faculty of Science, Cairo University, Cairo, Egypt; 2 Chemistry Department (Biochemistry Division), Faculty of Science, Cairo University, Cairo, Egypt; 3 Medical Biochemistry and Molecular Biology Department, Faculty of Medicine, Ain Shams University, Cairo, Egypt; 4 Translational & Applied Science Hub (TASH), Faculty of Medicine, Ain Shams University, Cairo, Egypt

**Keywords:** Acute kidney injury, Acute coronary syndrome, *GTF2I*, *ANGPTL4*, *MMP14*, Biomarkers

## Abstract

**Background:**

Acute kidney injury (AKI) represents a critical complication in patients with acute coronary syndrome (ACS), particularly in patients undergoing percutaneous coronary intervention (PCI). Current tests for detecting early AKI, such as creatinine and cystatin C, have modest sensitivity. This study explores the role of bioinformatics in creating an implementable predictive key gene in cardiorenal pathogenesis and evaluates the diagnostic potential of *GTF2I*,* ANGPTL4*, and *MMP14* in predicting AKI in ACS patients.

**Methods:**

This is a single-center prospective observational cohort study that enrolled 167 participants: healthy controls (*n* = 60), ACS patients (*n* = 56), and ACS patients with AKI (*n* = 51). Baseline clinical and renal markers were measured 24 h pre-PCI, with RNA biomarkers (*GTF2I*, *ANGPTL4*, *MMP14*) assessed at 48 h post-PCI, with daily creatinine monitoring performed for up to 6 days after PCI.

**Results:**

*GTF2I*,* ANGPTL4*, and *MMP14* were significantly elevated in ACS patients, with further increases in ACS-AKI patients. *GTF2I* showed the strongest predictive accuracy for AKI (AUC = 0.98), outperforming creatinine and cystatin. *ANGPTL4* correlated negatively with serum creatinine (*r* = − 0.213, *P* = 0.006), suggesting a modulatory role in renal injury, while *MMP14* elevations were pronounced in ACS-AKI. Notably, the combined biomarker panel achieved the highest diagnostic accuracy (AUC = 0.99), underscoring its potential as a robust early predictor of AKI. Subgroup analysis in diabetic patients revealed differential biomarker expression between AKI and non-AKI cases. Logistic regression confirmed albuminuria, *ANGPTL4*, *GTF2I*, and *MMP14*, in addition to several metabolic biomarkers, as significant predictors of AKI.

**Conclusion:**

*GTF2I*, *ANGPTL4*, and *MMP14* are promising biomarkers for predicting AKI in ACS patients and could be integrated into clinical practice for early AKI detection, improving patient management and outcomes.

**Supplementary Information:**

The online version contains supplementary material available at 10.1186/s12882-026-04926-w.

## Introduction

Acute coronary syndrome (ACS), despite ample progress in its diagnosis and management, remains a global burden, linked to a considerable proportion of worldwide mortalities [1]. Almost 50% of these deaths can be directly attributed to ischemic heart disease [2]. ACS is classified based on electrocardiographic (ECG) findings into ST-segment elevation myocardial infarction (STEMI) and non-ST-segment elevation ACS (NSTE-ACS) [3]. NSTE-ACS is further subdivided into non-ST-segment elevation myocardial infarction (NSTEMI) and unstable angina (UA), contingent upon whether or not myocardial necrosis is present [4].

Acute kidney injury (AKI) is a common and critical complication following cardiac surgery [5, 6]. It is estimated that up to 30% of patients with ACS develop AKI, contributing to approximately 2 million deaths annually worldwide [7, 8]. AKI is hallmarked by a sudden impairment of renal function, commonly detected as a rise in serum creatinine of ≥ 0.3 mg/dL [8]. There is significant inconsistency in the management of AKI, largely attributed to insufficient awareness and the lack of standardized guidelines for its prompt identification and treatment [9, 10]. Recognized globally as a “silent killer”, AKI has recently garnered significant attention in clinical research [10]. Efforts to explain the underlying causes of AKI must be taken with a new urgency, as delayed recognition in cardiac surgery patients may thwart preventive measures and result in irreversible kidney damage.

Serum creatinine is commonly used as the gold standard for assessing AKI in patients with ACS [11]. However, despite its long-standing use, creatinine is not an ideal biomarker and is prone to inaccuracies [12]. Its levels are influenced by various factors, including an individual’s muscle mass, dietary protein intake, liver function, and age, particularly as muscle mass decreases with aging [13]. Additionally, depending solely on creatinine for diagnosing AKI has its limitations, since significant fluctuations in creatinine levels may not be observable for up to 48 h [14]. Cystatin C is widely recognized as a viable alternative biomarker for kidney function that offers superior predictions of mortality and cardiovascular incidents when compared to creatinine levels; however, its interpretation in individuals with AKI, chronic kidney disease (CKD), and end-stage kidney disease has led to some diagnostic indeterminacy [15–17].

Nevertheless, understanding the pathogenesis of ACS-AKI is crucial for identifying early diagnostic and decisive prognostic biomarkers. The underlying mechanisms of ACS-AKI are complex and remain elusive [18]. However, advancements in omics technologies, such as transcriptomics, which measures mRNA abundance in cells or biofluids, have created new avenues for the non-invasive diagnosis of AKI [18, 19].

Recent studies suggest that early alterations in lipid metabolism are implicated in AKI development [20]. AKI triggers a remarkable transformation in the metabolic processes of renal tubular epithelial cells, affecting lipid, glucose, and amino acid metabolism. Changes in lipid metabolism encompass both a reduction in fatty acid oxidation and alterations in the lipids of cell membranes and triglyceride metabolism [21]. Disrupted intracellular lipid buildup can interfere with autophagic clearance, resulting in additional lipid deposition and diminished autophagic activity, thus establishing a self-perpetuating cycle [22].

Moreover, angiogenic markers are increasingly recognized as pivotal in the pathophysiology and outcomes of AKI, with evidence indicating that angiogenesis significantly influences kidney recovery and long-term prognosis [22, 23]. Mansour et al. demonstrated that the balance between pro- and anti-angiogenic factors is crucial in dictating the trajectory of kidney recovery [24]. Moreover, enhanced neovascularization can aid in preserving peritubular capillaries, mitigating tubulointerstitial fibrosis, and stabilizing renal function following injury [24].

General transcription factor IIi (*GTF2I*) plays a role in neurocognitive disorders, systemic lupus erythematosus (SLE), and cancer metastasis [25]. *GTF2I* polymorphisms have been linked to the severity of kidney impairment in SLE, particularly lupus nephritis (LN). These polymorphisms are associated with increased markers of kidney damage, including proteinuria, elevated creatinine, and inflammatory cytokines. Additionally, *GTF2I* variants correlate with the presence of nephrotoxic autoantibodies (anti-dsDNA, anti-Sm, and anti-RNP), which contribute to kidney inflammation and dysfunction in LN [26].

The angiopoietin-like family, including *ANGPTL4*, is crucially involved in key physiological and pathological processes such as angiogenesis, inflammation, and the regulation of lipid, glucose, and energy metabolism [27]. Elevated *ANGPTL4* levels have been linked to dyslipidemia, metabolic syndrome traits, and cardiovascular diseases like ACS and heart failure [27, 28]. *ANGPTL4* is also a key biomarker in chronic hemodialysis patients, with significantly higher levels observed, and it correlates independently with renal function. In diabetic nephropathy (DN), *ANGPTL4* is upregulated earlier than albuminuria and podocyte injury, indicating its possible role as a diagnostic and treatment marker for kidney damage [29, 30].

Matrix metalloproteinases (MMPs) represent an essential family of proteins that participate in diverse physiological functions, such as promoting new blood vessel formation, inflammation, and facilitating vascular repair [31]. Among these, *MMP14*, a membrane-type *MMP*, plays a pivotal role in vascular health by facilitating endothelial cell migration, extracellular matrix degradation, and capillary tube formation [32]. *MMP14*’s activity is linked to various vascular pathologies, such as atherosclerosis, hypertension, and plaque vulnerability [33]. Moreover, it is usually upregulated in kidney injury disease [34].

Based on the established roles of *ANGPTL4* in metabolic dysregulation and angiogenic signaling, *GTF2I* in inflammatory responses, and *MMP14* in vascular remodeling and tissue injury, we hypothesized that the expression of these biomarkers would be significantly increased in ACS patients who develop AKI compared with those who do not.

Accordingly, this study aims to evaluate the diagnostic potential of serum biomarkers *GTF2I*, *ANGPTL4*, and *MMP14* in patients with ACS, focusing on their ability to predict AKI following PCI. Furthermore, the study examines the performance of these biomarkers in comparison to established clinical scores.

## Materials and methods

### Sample size calculation

Sample size was calculated a priori using a two-proportion z-test, powered to detect a clinically meaningful difference in AKI incidence between our prospective cohort and the published baseline rate. Based on prior literature, AKI incidence following primary PCI in ACS patients has been reported at approximately 27% [35]. We anticipated a higher rate in our cohort given the inclusion of both STEMI and NSTEMI patients, with a broad spectrum of risk factors, including high prevalences of diabetes and dyslipidemia, estimating an expected incidence of approximately 45%. Using a two-sided alpha of 0.05 and a power of 80%, the required sample size was calculated as 108 ACS patients (formula: n = [(zα√(p̂(1−p̂)) + zβ√(p̂(1−p̂))) / (p₁ − p₀)]², where p₀ = 0.27 and p₁ = 0.45). An additional 60 healthy volunteers were enrolled as a reference group to establish normal expression ranges for the novel RNA biomarkers (*GTF2I*,* ANGPTL4*,* MMP14*), yielding a total planned enrolment of 168 participants. A total of 167 participants were ultimately enrolled and included in the final analysis (ACS without AKI: *n* = 56; ACS with AKI: *n* = 51; healthy controls: *n* = 60), which met the pre-specified minimum. The observed AKI incidence was 47.6%, consistent with our a priori assumption. Retrospective power analysis at the observed primary diagnostic AUC of 0.98 (*GTF2I*, ACS-AKI vs. ACS) confirmed that the enrolled sample provided > 99% statistical power for the primary biomarker comparison.

### Study population

This was a single-center prospective observational cohort study. The study population was recruited from the Cardiovascular Department at Ain Shams University Hospital between January 2024 and September 2024 with signs of acute myocardial infarction (AMI). Consent with full information was acquired from all participants before they were enrolled, and the study was granted ethical approval by the Ain Shams University Faculty of Medicine Ethics Committee (FWA 000017585), adhering to the Declaration of Helsinki guidelines. A total of 167 participants were included, with comprehensive data collected on their demographic and clinical characteristics.

ACS was diagnosed according to the 2023 ESC/ACC/AHA/WHF Fourth Universal Definition of Myocardial Infarction, requiring ischemic chest pain within six hours, elevated hs-cTnI (high-sensitivity cardiac troponin I) and CK-MB levels, and pathological ECG changes such as Q waves or ST-segment alterations [36]. Following diagnosis, all patients underwent a comprehensive cardiac assessment, including an ECG for ischemic changes and echocardiography to evaluate ventricular function.

The primary endpoint was the development of AKI, defined according to Kidney Disease: Improving Global Outcomes (KDIGO) guidelines as an increase in serum creatinine ≥ 0.5 mg/dL within 48 h of PCI or a ≥ 50% rise from baseline within seven days of the procedure [37]. Eligible participants had their creatinine levels monitored from the day before the procedure up to six days post-PCI. The cohort was divided into the following subgroups:

1. Healthy Control Group (*n* = 60): Healthy volunteers with normal ECG and no history of cardiovascular or renal diseases.
2. ACS Group (*n* = 56): Patients diagnosed with ACS but did not develop AKI within the study period.
3. ACS-AKI Group (*n* = 51): ACS patients who developed AKI within 7 days of PCI.

**Fig. 1:**
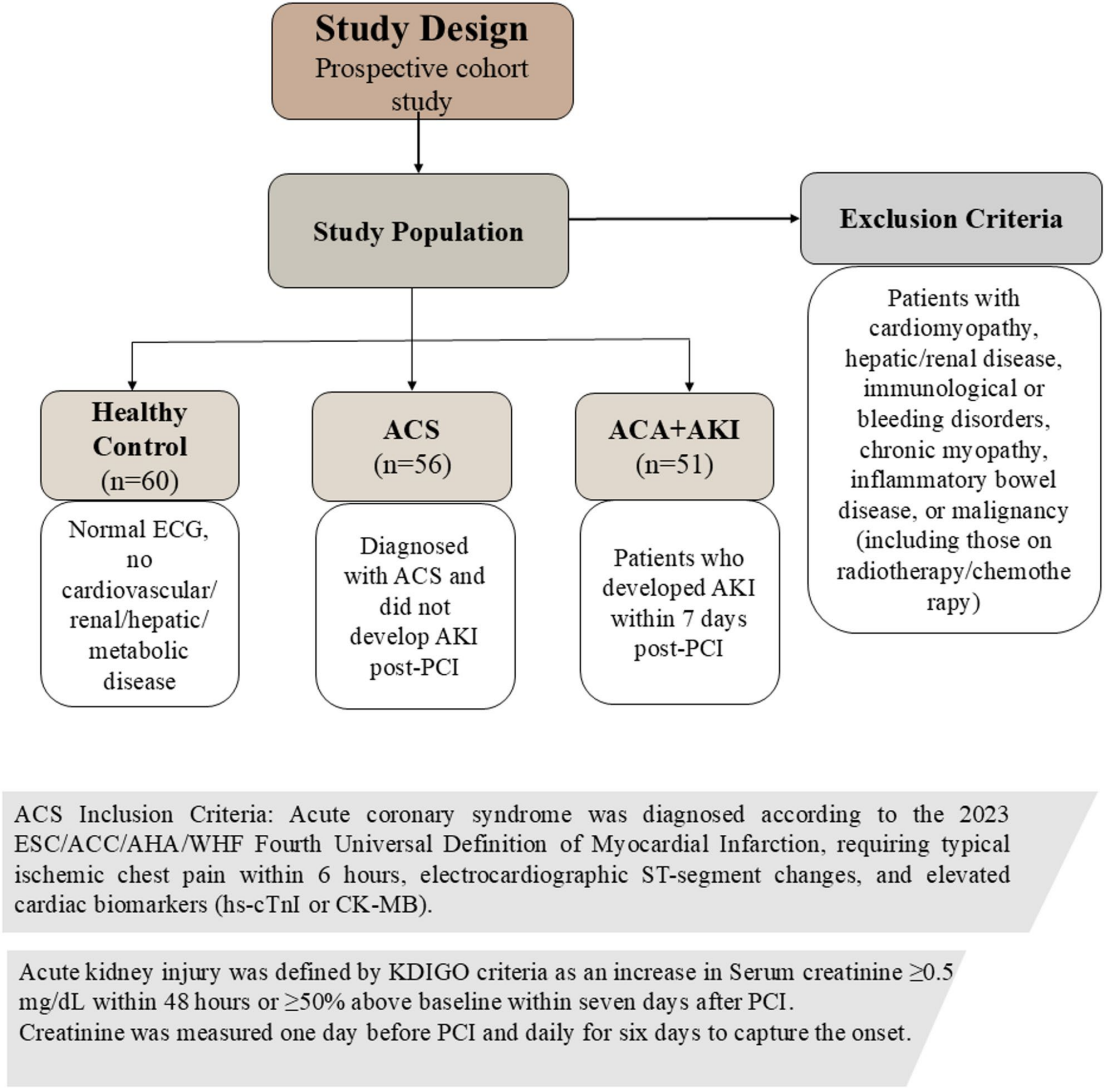
Flow diagram of study design. Abbreviations: ACS, acute coronary syndrome; AKI, acute kidney injury; PCI, percutaneous coronary intervention; ECG, electrocardiography; CK-MB, creatine kinase-MB; KDIGO, Kidney Disease: Improving Global Outcomes

The study’s exclusion criteria included patients with cardiomyopathy, hemorrhagic or immunological disorders, hepatic and renal diseases, inflammatory bowel disease, or chronic myopathy. Patients with cancer or undergoing radiotherapy or chemotherapy were also excluded. Additionally, patients with renal transplants or receiving dialysis were not eligible for this study (Fig. 1).

### Biochemical assessment of cardiac and renal biomarkers

Blood and urine specimens were obtained from all study participants at predefined time points. Samples were centrifuged at 4000 rpm for 20 min, and the separated serum was aliquoted and stored at − 80 °C in DNase-/RNase-free eppendorf tubes until analysis. Baseline serum samples collected 24 h prior to PCI were used for routine biochemical measurements, including Alanine Aminotransferase (ALT), Aspartate Aminotransferase (AST), Total Cholesterol (TC), Low-Density Lipoprotein cholesterol (LDLc), High-Density Lipoprotein cholesterol (HDLc), Triglycerides (TG), Hemoglobin A1c (HbA1c), cystatin C, Insulin, Glucose, and Estimated Glomerular Filtration Rate (eGFR) were determined using a multifunctional automated biochemistry analyzer (AU680, Beckman Coulter Inc., Brea, CA, USA). ACS diagnosis required elevated hs-cTnI levels above the 99th percentile upper reference limit (≥ 34 ng/L for men and ≥ 16 ng/L for women), with age- and sex-adjusted thresholds as per assay specifications [38–40]. eGFR was calculated using the Chronic Kidney Disease Epidemiology Collaboration (CKD-EPI) creatinine Eq. (2009), without application of the race coefficient, in accordance with National Institute of Diabetes and Digestive and Kidney Diseases (NIDDK) recommendations [41–43].

A serum sample collected 48 h after PCI was used for molecular analysis of *GTF2I*, *ANGPTL4*, and *MMP14* gene expression.

### In silico analysis

The Gene Expression Omnibus (GEO) database (www.ncbi.nlm.nih.gov/geo, accessed January 2024), a public repository for high-throughput gene expression data, was utilized for this study. The search was limited to the keywords “Acute Coronary Syndrome” and “Acute Kidney Injury or chronic kidney disease”. Only data for Homo sapiens were considered, and the selection criteria included expression profiling by array, tissue, or blood samples from both diseased and normal subjects, and datasets with adequate sample sizes to ensure sufficient statistical power. Three datasets: GSE36895, GSE19339, and GSE116626 were ultimately selected based on their relatively large sample sizes (*n* > 5) and comprehensive sample information [35, 36]. Differentially expressed genes (DEGs) were identified using GEO2R and the R package limma, with a significance threshold of *P*-value < 0.05 and |logFC| > 0.5 (Tables S1 & S2). Three genes, *MMP14*, *GTF2I*, and *ANGPTL4*, were selected for their roles in lipid metabolism and angiogenesis. Protein-protein interaction and Gene Ontology analysis were performed using the GeneMANIA database (https://genemania.org/, accessed January 2024) (Fig. S1), ShinyGO 0.80 (http://bioinformatics.sdstate.edu/go/, accessed January 2024) (Fig. S2), and GeneCards databases (https://www.genecards.org/, accessed January 2024), respectively (Fig. S3).

### Quantitative real-time PCR

**Fig. 2:**
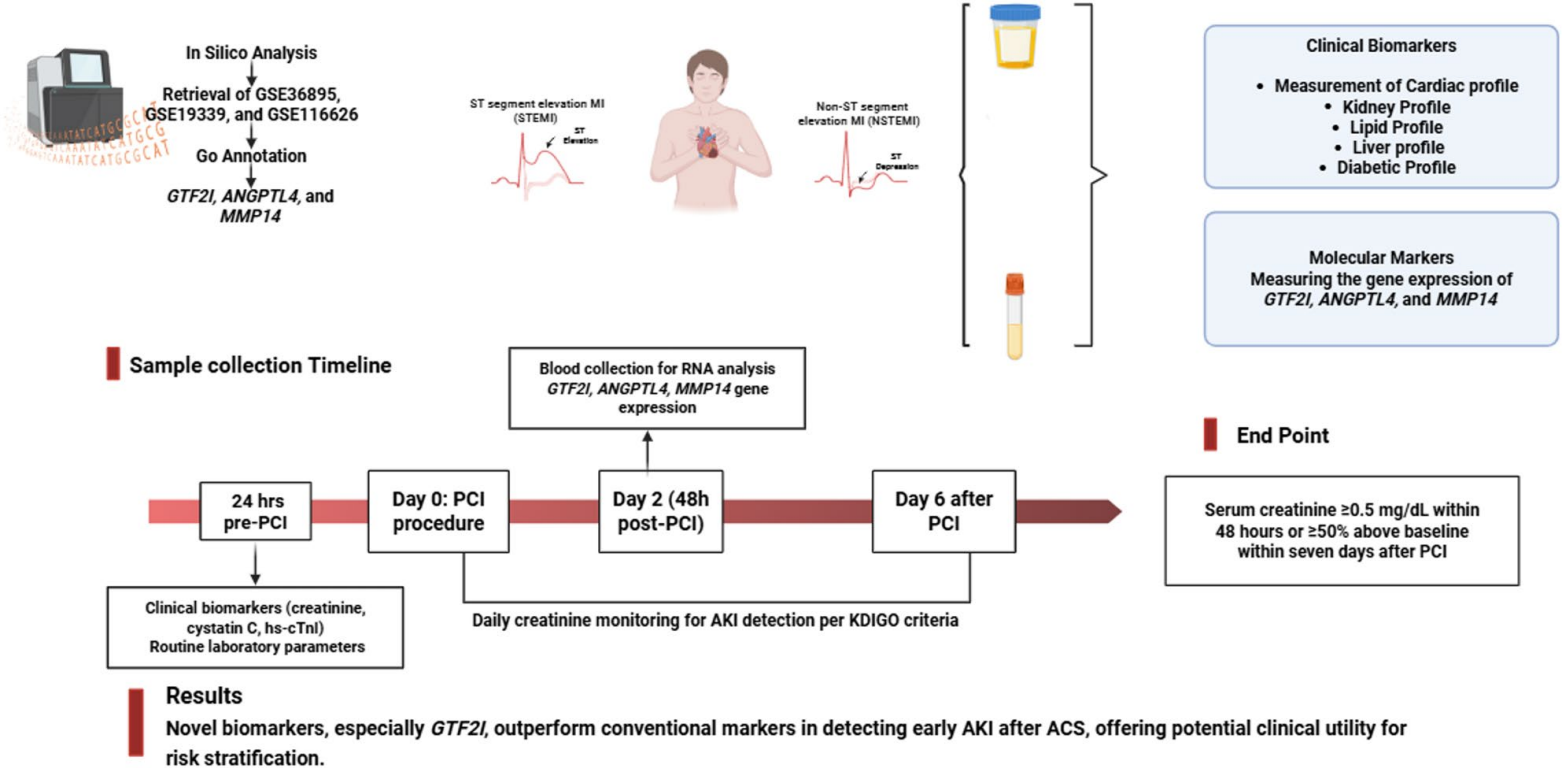
Graphical abstract illustrating the overall study design. Abbreviations: PCI, percutaneous coronary intervention, hs-cTnI, High-sensitivity cardiac troponin I

Total RNA was extracted from serum samples (200 µl) using the RNeasy Maxi Kit (Cat. No. 75162, Qiagen, Valencia, CA), according to the manufacturer’s protocol. Serum samples were lysed using Buffer RLT containing β-mercaptoethanol. The flow-through was mixed with ethanol and applied to RNeasy Mini spin columns for RNA binding. After washing steps with Buffer RW1 and Buffer RPE, total RNA was eluted in 30 µl RNase-free water. RNA quantity and purity were evaluated with a NanoDrop spectrophotometer (Thermo Scientific, Waltham, MA, USA). Samples with an A260/A280 ratio between 1.8 and 2.1 were deemed suitable for further analysis. Additionally, RNA concentration was verified using the Invitrogen™ Qubit™ 3.0 Fluorometer (Thermo Scientific, Waltham, MA, USA). For reverse transcription, 500 ng of total RNA was used for complementary DNA (cDNA) synthesis with the QuantiTect Reverse Transcription Kit (Cat no. / ID. 205311, Qiagen, Hilden, Germany) on a Rotor-Gene Thermal Cycler (Thermo Electron, Waltham, MA, USA). Relative quantification of target mRNAs was carried out using the QuantiTect SYBR Green PCR Master Mix (Qiagen, Cat. No. 204143). The expression levels of the target genes *ANGPTL4* (Accession No. PM00191611), *GTF2I* (Accession No. SBH0173388), and *MMP14* (Accession No. PM00057190) were quantified using QuantiTect Primer Assays (Qiagen, Hilden, Germany). Gene expression levels were normalized to the housekeeping gene *GAPDH* (Accession No. SBH0555552), which served as the internal control. All primer assays, kits, and reagents used in the quantitative PCR analysis were obtained from Qiagen, Germany. Data were analyzed using the 2^–ΔΔCt^, following the approach outlined by Livak [44]. All kits complied with the manufacturer’s specifications. Amplification and quantification were conducted on the Applied Biosystems 7500 FAST Real-Time PCR System (Applied Biosystems, Foster City, CA, USA). The overall study framework is summarized in Fig. 2.

### Statistical analysis

Statistical analyses were performed using SPSS version 25.0 (SPSS Inc., Chicago, IL, USA). The Shapiro-Wilk test was used to assess the normality of distribution for continuous variables. Variables with a *P*-value > 0.05 on the Shapiro-Wilk test were considered normally distributed (reported as mean ± SD and compared with ANOVA followed by Tukey’s post-hoc test), while non-normally distributed variables were reported as median (IQR) and compared using the Kruskal-Wallis test with Dunn’s correction for multiple comparisons. Categorical variables were compared using the Chi-square test. Correlations between biomarkers and clinical measures were assessed with Spearman’s coefficient, diagnostic accuracy was evaluated by ROC curves, and independent predictors of AKI were determined using multivariable logistic regression. To identify independent predictors of AKI, we performed binary logistic regression analysis. Variable selection for the multivariable model was guided by a two-step process. First, a univariate screening of all relevant clinical and molecular variables was performed using the Score test. Variables demonstrating a significant association with AKI (*P* < 0.05) in this univariate analysis, along with those of strong established clinical relevance, were considered for inclusion in the final multivariable model to avoid overfitting. The final model aimed to be parsimonious and included albuminuria categories, diabetes duration, and the three RNA biomarkers (*ANGPTL4*,* GTF2I*,* MMP14*).

This study was conducted and reported in accordance with the TRIPOD (Transparent Reporting of a multivariable prediction model for Individual Prognosis or Diagnosis) guidelines to ensure methodological transparency and completeness.

## Results

### Baseline characteristics across patient groups

A total of 107 patients with ACS were included in this study, who had blood tests and follow-up data and underwent PCI. Table 1 outlines the baseline characteristics of the cohort. For the demographic features, there was no significant change in the gender distribution (Chi-square test: *P* = 0.737), while the mean age was significantly higher in the ACS-AKI group compared to the healthy controls (one-way ANOVA: *P* = 0.038). BMI was significantly elevated in both ACS and ACS-AKI groups compared to healthy controls (Kruskal-Wallis test: *P* < 0.001), and the proportion of current smokers was significantly higher in both patient groups (Chi-square test: *P* < 0.001). A significant proportion of the ACS and ACS-AKI groups had a positive family history of cardiovascular disease (83.9% and 88.2%, respectively) (Chi-square test: *P* < 0.05). There were significant differences in the prevalence and duration of diabetes mellitus among patient groups (Chi-square test: *P* < 0.001). All healthy controls were non-diabetic, while diabetes was highly prevalent among ACS and ACS-AKI patients. Markers of glucose metabolism, including fasting and postprandial glucose levels and HbA1c, were significantly elevated in both patient groups compared to healthy controls (Kruskal-Wallis test: *P* < 0.001). The highest values were observed in the ACS-AKI group, indicating poor glycemic control. Insulin resistance, as assessed by HOMA-IR, was markedly increased in both ACS groups, while HOMA-B was significantly reduced, particularly in the ACS-AKI group (Kruskal-Wallis test: *P* < 0.001). Both systolic and diastolic blood pressures were significantly higher in ACS and ACS-AKI patients compared to controls (Kruskal-Wallis test: *P* < 0.001). Lipid profiles revealed pronounced dyslipidemia in the patient groups. Total cholesterol, LDL cholesterol, and triglycerides were significantly elevated in both ACS groups, while HDL cholesterol was markedly reduced, with the lowest levels observed in the ACS-AKI group (Kruskal-Wallis test: *P* < 0.001). ALT levels peaked at the ACS group, while AST levels were significantly higher in both ACS and ACS-AKI groups compared to controls (Kruskal-Wallis test: *P* < 0.001).

**Table 1:** Baseline demographic and clinical characteristics of the participants

Variables	Healthy control *n* = 60	ACS *n* = 56	ACS-AKI *n* = 51	*P*-value
Age, year, Mean ± SD	51.95 ± 7	53.95 ± 8.5	55.78 ± 7.9^a^	0.038
BMI, kg, median (IQR)	33(18–39)	36(19–44)^a^	34.7(22.5–45)^a^	0.000
Gender				
Male, n (%)	22(36.7%)	19(33.9%)	21(41.2%)	0.737
Female, n (%)	38(63.3%)	37(66.1%)	30(58.8%)	
Smoking				0.000
Non-smoker, n (%)	51(85%)	11(19.6%)	16(31.4%)	
Smoker, n (%)	8(13.3%)	43(76.8%)	30(58.8%)	
X-Smoker, n (%)	1(1.7%)	2(3.6%)	5(9.8%)	
Family History				0.000
+Ve, n (%)	-	47(83.9%)	45(88.2%)	
-Ve, n (%)	60(100%)	9(16.1%)	6(11.8%)	
Diabetes Mellitus				0.000
Not Diabetic, n (%)	60(100%)	1(3.7%)	-	
< 5 years, n (%)	-	6(22.5%)	-	
5–10 years, n (%)	-	13(48.1%)	19(37.3%)	
> 10 years, n (%)	-	7(45.9%)	32(62.7%)	
Fasting Glucose, mg/dl, median (IQR)	88(70–125)	110(73–400)^a^	180(100–394)^ab^	0.000
Post Prandial Glucose, mg/dl, median (IQR)	109(80–138)	180(97–490)^a^	270(196–620) ^ab^	0.000
HbA1c (%), median (IQR)	4(2-5.5)	5(3-9.95)^a^	9(4–17) ^ab^	0.000
HOMA-IR, median (IQR)	0.64(0.09–2.36)	4.98(0.44–18.4)^a^	5.05(1.26–11.9)^a^	0.000
HOMA-B (%), median (IQR)	202.5(160–240)	88(44–221)^a^	51(40–67)^ab^	0.000
Systolic blood pressure, mmHg, median (IQR)	120(100–130)	140(90–170)^a^	130(120–160)^a^	0.000
Diastolic blood pressure, mmHg, median (IQR)	80(70–85)	90(50–110)^a^	90(70–110)^a^	0.000
Total Cholesterol, mg/dl, median (IQR)	95(70–150)	270(200–400)^a^	300(160–400)^a^	0.000
LDL, mg/dl, median (IQR)	70(50–120)	190(100–300)^a^	190(84–300)^a^	0.000
HDL, mg/dl, median (IQR)	67(55–90)	42(23–60)^a^	30(20–45)^ab^	0.000
TGs, mg/dl, median (IQR)	100(80–130)	240(125–350)^a^	300(123–400)^ab^	0.000
ALT, U/L, median (IQR)	38.5(18–91)	47(26–91)^a^	40(26–69)^b^	0.01
AST, U/L, median (IQR)	38(19–77)	48.5(19–94)^a^	51(33–358)^a^	0.000

^a^*P*< 0.05 compared with the control group, ^b^*P*<0.05 compared with the ACS group. Abbreviations: ACS, acute coronary syndrome; AKI, acute kidney injury; PCI, percutaneous coronary intervention; BMI, body mass index; HbA1c, glycated hemoglobin A1c; HOMA-IR, homeostatic model assessment of insulin resistance; HOMA-B, homeostatic model assessment of β-cell function; LDL, low-density lipoprotein; HDL, high-density lipoprotein; TGs, triglycerides; ALT, alanine aminotransferase; AST, aspartate aminotransferase

### Comparative analysis of renal and cardiac biomarkers among study groups

Cystatin C levels were significantly elevated in both ACS patients without AKI (median: 1.028 mg/L, IQR: 0.6–1.98) and patients with AKI (median: 1.03 mg/L, IQR: 0.74–1.4) compared to healthy controls (median: 0.93 mg/L, IQR: 0.72–1.1) (Kruskal-Wallis test: *P* < 0.001). While the median creatinine level in the ACS group without AKI (0.79 mg/dL, IQR: 0.65–0.91) was slightly lower than in healthy controls (0.8 mg/dL, IQR: 0.7–1.2), patients in the ACS-AKI group exhibited a broader range of creatinine values (median: 0.8 mg/dL, IQR: 0.65–1.89), which was significantly different from ACS groups (Kruskal-Wallis test: *P* = 0.003). Normoalbuminuria was observed in control and ACS without AKI groups, whereas nearly 50% of patients in both AKI subgroups developed macroalbuminuria (Chi-square test: *P* < 0.001). eGFR declined progressively from healthy individuals to ACS patients without AKI, and was lowest in those with ACS-AKI patients (Kruskal-Wallis test: *P* < 0.001). The Alb/creat ratio was significantly elevated in both ACS groups and more significantly in ACS-AKI patients (Kruskal-Wallis test: *P* < 0.001). Cardiac markers showed substantial elevations in both ACS groups. CK-MB concentrations were significantly higher in ACS patients with and without AKI compared to healthy individuals (Kruskal-Wallis test: *P* < 0.001). Troponin levels followed the same pattern, with significantly greater elevations in ACS patients, particularly those who developed AKI (Kruskal-Wallis test: *P* < 0.001) (Table 2).

**Table 2:** Comparison of renal and cardiac biomarkers between healthy controls, ACS patients without AKI, and ACS patients with AKI

Variables	Healthy control *n* = 60	ACS *n* = 56	ACS-AKI *n* = 51	*P*-value
cystatin C, mg/L, median (IQR)	0.93(0.72–1.1)	1.028(0.6–1.98)^a^	1.03(0.74–1.4)^a^	0.000
Creatinine, mg/dl, median (IQR)	0.8(0.7–1.2)	0.79(0.65–0.91)^a^	0.8(0.65–1.89)^b^	0.003
Albuminuria				0.000
Normoalbuminuric, n (%)	60(100%)	56(100%)	-	
Microalbuminuric, n (%)	-	-	26(51%)	
Macroalbuminuric, n (%)	-		25(49%)	
eGFR, ml/min, Mean ± SD	103.83(90–123)	94(88–102)^a^	59(30–90)^ab^	0.000
Alb/creat ratio, mg/g, median (IQR)	13(9–21)	23(13–30)^a^	26(18–33)^a^	0.000
CK-MB, U/L, median (IQR)	6.15(1–23)	33(0.6–135)^a^	34(4.5–113)^a^	0.000
hs-cTnI, ng/L, median (IQR)	0.41(0.001–4.84)	5.38(0.08–100)^a^	33(0.01-23)^ab^	0.000

^a^*P*< 0.05 compared with the control group, ^b^*P*<0.05 compared with the ACS group. ACS acute coronary syndrome, AKI acute kidney injury, PCI percutaneous coronary intervention, eGFR estimated glomerular filtration rate, Alb/creat ratio albumin-to-creatinine ratio, CK-MB creatine kinase-MB, hs-cTnI High-sensitivity cardiac troponin I

### Differential expression and correlation analysis of biomarkers

The differential expression analysis of serum markers *GTF2I*,* ANGPTL4*, and *MMP14* by the Kruskal-Wallis test with Dunn’s post-hoc analysis demonstrated notable increases across all groups related to ACS when compared to healthy controls (*P* < 0.0001), and this increase was more pronounced after the AKI onset. Levels of *GTF2I* increased significantly across both ACS and ACS-AKI by 3.3 and 3.58-fold, respectively, compared to the control group. Similarly, *ANGPTL4* peaked by 3.38-fold in the ACS-AKI group compared to the control group. Additionally, the *MMP14* exhibited the same pattern and increased significantly in the ACS-AKI group by 2.23-fold (Table 3 & Fig. S4).

Importantly, stratification by diabetic status revealed a distinct molecular signature associated with AKI development among diabetic patients. Within this subgroup, *ANGPTL4* and *MMP14* expression levels were significantly higher in patients who developed AKI than in diabetic patients with ACS alone (*P* < 0.05). Conversely, *GTF2I* expression did not differ significantly between diabetic patients with ACS-AKI and those with ACS (Table 4).

Spearman’s correlation analysis revealed significant relationships between the biomarkers *GTF2I*, *ANGPTL4*, and *MMP14* and clinical indicators of cardiac injury, specifically CK-MB and Troponin. *GTF2I* exhibited a strong positive correlation with CK-MB (*r* = 0.545, *P* < 0.001) and Troponin (*r* = 0.547, *P* < 0.001), along with *ANGPTL4* (*r* = 0.489, *P* < 0.001) and *MMP14* (*r* = 0.435, *P* < 0.001). *ANGPTL4* was also positively correlated with CK-MB (*r* = 0.567, *P* < 0.001) and Troponin (*r* = 0.492, *P* < 0.001), showing a moderate correlation with MMP14 (*r* = 0.383, *P* < 0.001). *MMP14*, in turn, demonstrated positive correlations with both CK-MB (*r* = 0.396, *P* < 0.001) and Troponin (*r* = 0.432, *P* < 0.001). Notably, *ANGPTL4* displayed a significant negative correlation with serum creatinine (*r* = -0.213, *P* = 0.006), while no significant correlations were found between *GTF2I* or *MMP14* and serum creatinine. Cystatin C displayed moderate positive correlations with CK-MB (*r* = 0.226, *P* < 0.01) and troponin (*r* = 0.373, *P* < 0.01), while it had a weak negative correlation with serum creatinine (*r* = -0.203, *P* < 0.01). These correlations suggest that elevated levels of these biomarkers are associated with increased cardiac injury markers and impaired renal function (Table S3).

**Table 3:** Differential Expression Analysis of Serum *GTF2I*,* ANGPTL4*, and *MMP14* across study groups

Variables	Healthy control	ACS	ACS-AKI	*P*-value
*GTF2I*, median (IQR) Mean Rank	0.78(0.11–2.5) 32.76	7.27(0.4-17.98)^a^ 108.54	7.8 (1-117)^a^ 117.34	0.000
*ANGPTL4*, median (IQR) Mean Rank	1(0.047–3.92) 41.78	2.45(0.26–34.8)^a^ 76.88	34.92(14.7-120.1)^ab^ 141.49	0.000
*MMP14*, median (IQR) Mean Rank	5.65(0.01–98.3) 53.06	33.25(2.1-170.8)^a^ 85.99	177.25(2.39-5244.43)^ab^ 118.22	0.000

^a^*P* < 0.05 compared with the control group, ^b^*P*<0.05 compared with the ACS group. Abbreviations: ACS, acute coronary syndrome; AKI, acute kidney injury; PCI, percutaneous coronary intervention

**Table 4:** Comparison of *ANGPTL4*, *GTF2I*, and *MMP14* expression levels between ACS and ACS-AKI patients with diabetes mellitus

Variables	ACS	ACS-AKI	*P*-value
*GTF2I*, median (IQR) Mean Rank	7.27(0.4-13.42) 34.02	6.5(0.85–117) 41.54	0.163
*ANGPTL4*, median (IQR) Mean Rank	3.9(0.26–34.8) 14.5	34.92(14.7-120.1)^a^ 51.49	0.000
*MMP14*, median (IQR) Mean Rank	41.52(3.15–170.8) 28.6	177.25(2.39-5244.43)^a^ 44.3	0.004

^a^*P* < 0.05 compared with the ACS group. Abbreviations: ACS, acute coronary syndrome; AKI, acute kidney injury; PCI, percutaneous coronary intervention

### Prediction accuracy of *GTF2I*, *ANGPTL4*, and *MMP14*

In ROC curve analysis, GTF2I demonstrated outstanding diagnostic accuracy in distinguishing both ACS and ACS-AKI patients from healthy controls, with AUCs of 0.975 and 1, respectively. *ANGPTL4* showed excellent performance in differentiating ACS-AKI from ACS patients (AUC = 0.984), outperforming both cystatin C and creatinine, which exhibited limited discriminative ability across all comparisons (AUCs < 0.71). Notably, the combined panel of *GTF2I*,* ANGPTL4*, and *MMP14* achieved high diagnostic performance in all comparisons, with AUCs up to 0.989, highlighting its strong potential as a superior multi-marker tool for early detection and differentiation of cardiovascular and renal injury (Table 5 & Fig. S5-S7).

Univariate screening analysis identified several variables significantly associated with AKI (Table S4). Among the clinical parameters, albuminuria categories, diabetes status, HbA1c, triglycerides, serum creatinine, and eGFR showed significant associations (*P* < 0.05). In addition, the RNA biomarkers *ANGPTL4*, *GTF2I*, and *MMP14* were also significantly associated with AKI risk. In contrast, demographic variables such as age, sex, smoking status, and BMI were not significantly associated with AKI in the univariate screening analysis (Table S4).

Following the univariate screening analysis, variables demonstrating statistical significance and clinical relevance were further evaluated using multivariable logistic regression. The logistic regression analysis revealed that both categories (1 and 2) of albuminuria showed extremely strong associations with AKI risk (*P* < 0.001), aligning with clinical expectations. *ANGPTL4’*s exceptionally high score (56.6) suggests a dominant role in AKI prediction. Both biomarkers, *GTF2I* and *MMP14*, were statistically significant (*P* < 0.01), supporting their inclusion in the multivariable model. Diabetes strong independent association (*P* < 0.001), reinforcing its clinical relevance. Overall Model Highly significant (*P* < 0.001), validating the collective predictive power of these variables (Table S5).

**Table 5:** ROC Curve Analysis for *GTF2I*,* ANGPTL4*, and *MMP14* and kidney biomarkers

Biomarkers	AUC (95% CI)	Cut-off point	Sensitivity	Specificity	*P*-value
ROC Curve Analysis for Biomarkers in Differentiating Healthy Controls from ACS Patients					
*GTF2I*	0.975(0.94-1)	2.8	96.4%	100%	0.000
*ANGPTL4*	0.799(0.71–0.89)	1.22	85.7%	68.3%	0.000
*MMP14*	0.731(0.64–0.83)	20.69	73.2%	70%	0.000
Combined (GTF2I, ANGPTL4, and MMP14)	0.982(0.955-1)	0.99	98.2%	96.7%	0.000
cystatin C	0.705(0.61–0.8)	0.96	71.4%	65%	0.000
Creatinine	0.655(0.56–0.75)	0.79	50%	63.3%	0.004
ROC Curve Analysis for Biomarkers in Differentiating Healthy Controls from AKI Patients					
GTF2I	1(0.96-1)	3.9	98%	91.7%	0.000
ANGPTL4	0.883(0.81–0.96)	0.89	88.2%	81.7%	0.000
MMP14	0.845(0.79–0.92)	19.8	74.5%	70%	0.000
Combined (*GTF2I*,* ANGPTL4*, and *MMP14*)	0.936(0.882–0.99)	0.5	92.2%	95%	0.000
cystatin C	0.69(0.59–0.79)	0.98	62.7%	70%	0.001
Creatinine	0.532(0.41–0.63)	0.81	39.2%	68.3%	0.679
ROC Curve Analysis for Biomarkers in Differentiating ACS from ACS-AKI Patients					
*GTF2I*	0.568(0.45–0.68)	6.4	51%	50%	0.227
*ANGPTL4*	0.984(0.96-1)	14.83	96.1%	96.4%	0.000
*MMP14*	0.733(0.62–0.84)	51.44	70.6%	76.8%	0.000
Combined (*GTF2I*,* ANGPTL4*, and *MMP14*)	0.989(0.97-1)	0.885	78.4%	96.4%	0.000
cystatin C	0.524(0.41–0.64)	1.028	52.9%	50%	0.67
Creatinine	0.658(0.55–0.76)	0.79	68.6%	50%	0.005

Abbreviations: AUC, Area Under the Curve; ACS, acute coronary syndrome; AKI, acute kidney injury; PCI, percutaneous coronary intervention

## Discussion

Among patients with ACS, the occurrence of AKI is substantial and closely linked to high healthcare costs, prolonged hospital stays, and increased mortality [45]. Chong et al. reported an AKI incidence of approximately 4% in elective PCIs, while the incidence nearly tripled in those admitted with NSTEMI or STEMI [38, 39]. Another study revealed that the incidence of AKI rose by 10–30% following primary PCI [46].

AKI is typically identified by elevated creatinine levels, though this marker tends to increase late in the progressive and deteriorative stages of the kidney damage [47]. Recently, cystatin C has served as a reliable marker for the early detection of AKI; however, its predictive accuracy varies widely in several studies [15, 48]. Predicting AKI in real-time could facilitate preventative actions such as modifying medications, optimizing blood flow, and enhancing long-term monitoring [49]. Angiogenesis is crucial for recovery after AKI, as it regulates pathways driving kidney damage [24]. Disrupted angiogenesis, combined with capillary loss and hypoxia, accelerates disease progression [50]. Experimental AKI models highlight that while tubular cells repair effectively, vascular restoration is limited, with vascular density reduced by 30–50% post-injury. This loss restricts oxygen delivery, triggering hypoxia, inflammation, and fibrosis [51].

The present study highlights the diagnostic utility of *GTF2I*, *ANGPTL4*, and *MMP14* in identifying ACS patients at risk of developing AKI.

We enrolled 107 ACS patients, evaluated their demographics, clinical comorbidities, and laboratory data, and found that the incidence of AKI in our cohort was 47.6% of ACS patients who underwent PCI had developed AKI.

The timing of RNA biomarker measurement was considered in accordance with emerging AKI biomarker recommendations. The KDIGO definition of AKI incorporates a serum creatinine rise of ≥ 0.5 mg/dL within 48 h of an index exposure as a primary diagnostic criterion [37]. Moreover, the Acute Disease Quality Initiative (ADQI) 23 Consensus suggests that injury biomarkers should be assessed early after the renal insult and that combining functional and damage biomarkers may enhance diagnostic accuracy [52]. Our sampling at 48 h post-PCI is consistent with this recommendation and also corresponds to the timeframe highlighted by ADQI 16 as the threshold distinguishing rapidly reversible AKI from persistent AKI, a stage associated with increased risk of progression to acute kidney disease (AKD) and CKD [53].

The differential expression analysis demonstrated that *GTF2I*, *ANGPTL4*, and *MMP14* levels were markedly elevated in ACS patients, especially in those who progressed to AKI. The strong discriminative performance of our RNA biomarker panel at this time point suggests its potential utility as an early risk-stratification tool within the AKD monitoring framework proposed by ADQI. Furthermore, the findings showed that *GTF2I* was strongly correlated with cystatin C and cardiac injury biomarkers, CK-MB and Troponin, suggesting *GTF2I’s* pivotal role in both myocardial ischemia and renal injury. Dakal et al. found that *GTF2I* expression was significantly elevated in renal cancer and correlates positively with genetic alterations such as copy number changes and missense mutations [54]. Another study identified potential SUMOylation sites in *GTF2I*, indicating that post-translational modification may regulate its activity under hypoxic stress, further reinforcing its possible role in cardiac dysfunction during ischemia [55]. In the setting of AKI, particularly following ischemic injury, its regulation of angiogenic factors like vascular endothelial growth factor (*VEGF*) is vital for kidney recovery. *GTF2I* specifically influences the *VEGFR2* (Kdr/Flk1) gene, which serves as the main mediator of *VEGF* signaling in endothelial cells, thereby promoting angiogenesis. When *GTF2I* is mutated or dysregulated, it can disrupt the activation of these pathways, potentially leading to insufficient vascular repair and extended renal dysfunction after ischemic damage [56].

Interestingly, *ANGPTL4* showed inverse correlation with serum creatinine, indicating a potential regulatory role in the dynamics of kidney injury. This observation aligns with its established role in lipid metabolism, vascular permeability, ischemic-reperfusion, metastasis, and inflammatory signaling within the kidney [47, 49, 57, 58].

Dewey et al. proved that variants in *ANGPTL4* were significantly associated with reduced plasma TGs, which also affects lipid metabolism and consequently reduces the risk of coronary heart disease [59]. *ANGPTL4* has been implicated in angiogenesis through activation of JAK signaling pathways [60]. Additionally, previous research suggested that *ANGPTL4* may have a protective effect in cardiovascular conditions, such as angiotensin II-induced atrial fibrosis, by regulating signaling pathways like PPARγ in cardiac fibroblasts [61].

This glycosylated protein consists of two main domains: the C-terminal fibrinogen-like domain (c*ANGPTL4*) and the N-terminal coiled-coil domain (n*ANGPTL4*). The c*ANGPTL4* domain contributes to vascular disruption by interacting with vascular-endothelial cadherin (VE-Cad) and integrin α5β1 on endothelial cells, while the n*ANGPTL4* domain binds to lipoprotein lipases (LPLs), inhibiting their activity, particularly in the regulation of hypertriglyceridemia [58].

Growing evidence indicates that *ANGPTL4* is involved in podocyte injury and the development of proteinuria in kidney diseases [62]. Previous studies have shown that increased secretion of high-isoelectric point *ANGPTL4* by podocytes contributes to proteinuria and foot process effacement in humans, as well as in experimental models of minimal change disease (MCD) [63]. *ANGPTL4* is highly upregulated in conditions like IgA nephropathy, cisplatin-induced acute kidney injury, and diabetic nephropathy, where it exacerbates kidney damage [51–53]. In CKD models, *ANGPTL4* was significantly elevated and positively correlated with markers of renal injury, supporting its role in promoting kidney damage, which is consistent with our results [54, 55].

*MMP14* expression peaked in the ACS-AKI group compared to both the healthy and ACS groups, and it was also significantly correlated with cystatin C and cardiac biomarkers. *MMP14* is involved in various cardiovascular and renal processes [64]. As a metalloproteinase involved in extracellular matrix remodeling and vascular injury, its elevation likely reflects structural and microvascular alterations accompanying AKI after PCI.

By breaking down the basal membrane surrounding vascular smooth muscle cells, *MMP14* facilitates their transformation into proliferative and migratory forms, contributing to plaque stabilization in arteries [65]. Its activity is linked to the development of atherosclerosis, coronary artery stenosis, and heart failure [66].

*MMP14* also plays a significant role in promoting cell migration, invasion, and angiogenesis, and its overexpression has been associated with cancer metastasis and kidney fibrosis [67]. Additionally, *MMP14’s* regulation of extracellular matrix and interaction with tissue inhibitors of metalloproteinases influence key processes such as inflammation, wound healing, and fibrosis, particularly in CKD [68]. Landolt et al. proved that *MMP14* plays a crucial role in the epithelial-to-mesenchymal transition in clear cell renal cell carcinoma (ccRCC), with elevated expression correlated with miR-34a, contributing to tumor progression, fibrosis, and poorer patient survival, making it a potential therapeutic target [69].

ROC analysis highlighted the diagnostic strength of our selected biomarkers. *GTF2I* demonstrated excellent accuracy in distinguishing ACS and ACS-AKI from controls (AUC = 0.975 and 1, respectively), while *ANGPTL4* excelled in differentiating ACS-AKI from ACS patients (AUC = 0.984). The combined biomarker panel (*GTF2I*,* ANGPTL4*, and *MMP14*) achieved the highest diagnostic performance across all comparisons (AUC up to 0.989).

The results also demonstrated that our biomarkers, *GTF2I*,* ANGPTL4*, and *MMP14*, outperformed traditional renal markers in discriminating ACS-AKI from ACS patients. While serum creatinine and cystatin C showed only modest predictive accuracy (AUC = 0.65 and 0.70, respectively), both are known late indicators of renal impairment. These findings suggest that our biomarkers capture early molecular changes associated with AKI pathogenesis, offering superior sensitivity and specificity compared to conventional markers.

The multivariable model demonstrates that combining RNA biomarkers (GTF2I, MMP14, ANGPTL4) with albuminuria and diabetes status improves AKI risk stratification, offering a framework for personalized interventions while addressing potential confounding through standardized adjustments.

CK-MB and Troponin, two well-established cardiac biomarkers, however, clinical decision-making in patients with renal diseases based on Troponin and CK-MB levels requires careful consideration, particularly regarding patient management and outcomes [70]. Existing research indicates that elevated Troponin levels in CKD patients do not always signal acute cardiac injury, as impaired clearance and potential re-expression of Troponin in uremic myopathic skeletal muscles can contribute to higher levels [71, 72]. While CK-MB mass testing offers high sensitivity and specificity for diagnosing myocardial infarction (MI), it can yield false positives in cases of severe musculoskeletal injury and may produce false negatives in late MI detection due to its early release characteristics [73]. The interpretation of cardiac biomarkers, especially in patients with renal disease or end-stage renal disease (ESRD), poses unique challenges [74]. Troponin levels in this case are often elevated, and studies suggest this can be a predictor of both short- and long-term mortality [75].

Diabetic patients are at significantly higher risk for AKI when hospitalized with ACS, which aligns with our findings [76]. This is demonstrated by the high proportion of diabetic patients with disease duration greater than five years in the ACS-AKI groups, along with elevated metabolic markers such as fasting glucose, postprandial glucose, and HbA1c, which showed substantial increases in the ACS-AKI group. Notably, subgroup analysis of the diabetic group showed that AKI development correlated with markedly increased levels of *ANGPTL4* and *MMP14*, while *GTF2I* levels remained unchanged. This finding suggests that, in the diabetic milieu, the onset of AKI is associated with heightened inflammatory responses and extracellular matrix remodeling. These observations are consistent with mechanistic studies indicating that hyperglycemia in type 2 diabetes may heighten the risk of AKI by triggering profibrotic and proinflammatory processes in the kidneys [77]. Research from animal studies also suggests that hyperglycemia may lead to mitochondrial dysfunction, cell death, and kidney damage [78].

Several biomarkers have been extensively studied for the early detection and risk stratification of acute kidney injury, including neutrophil gelatinase-associated lipocalin (*NGAL*), kidney injury molecule-1 *(KIM-1*), and interleukin-18 (*IL-18*) [79]. NGAL is one of the most extensively studied early AKI biomarkers, showing promise in various clinical settings, including cardiac surgery and contrast-induced AKI [80]. NGAL levels rise within 2–6 h after kidney injury, considerably earlier than serum creatinine, with reported AUC values ranging from 0.45 to 0.9 [81, 82]. KIM-1, a transmembrane protein upregulated in proximal tubular cells following ischemic or nephrotoxic injury, has demonstrated good diagnostic accuracy with AUC values of 0.70–0.85 in various AKI settings [79, 83, 84]. The superior performance of *GTF2I* (AUC = 0.98) and the combined biomarker panel (AUC = 0.99) in our study suggests that RNA-based biomarkers may offer advantages over protein-based markers. RNA biomarkers provide dynamic insight into cellular regulatory states, and their higher copy numbers enable enhanced biological signal detection [85].

Although NGAL and KIM-1 were not directly assessed in this study, the identified biomarkers—*ANGPTL4*, *GTF2I*, and *MMP14*—likely provide complementary information by reflecting metabolic dysregulation, inflammatory signaling, and extracellular matrix remodeling, processes that are particularly relevant in acute coronary syndrome–associated AKI. Future studies integrating RNA-based markers with established AKI biomarkers may further improve early risk prediction and clinical applicability.

This study has several limitations. First, the relatively small, single-center cohort (*n* = 167) limits generalizability. The exceptionally high diagnostic performance metrics (e.g., combined panel AUC = 0.99) and the strong univariate associations observed (e.g., albuminuria Score = 78.0, *P* < 0.001; *ANGPTL4* Score = 31.685, *P* < 0.001) are promising. However, they also raise a significant concern for potential model overfitting. Such performance is characteristic of discovery-phase studies and typically attenuates upon validation in independent, external cohorts. We did not perform internal validation techniques such as bootstrapping or cross-validation, and therefore, these results must be considered preliminary. Rigorous external validation in larger, multicenter, and diverse populations is an essential prerequisite before these biomarkers can be considered for routine clinical application. Second, we lacked comprehensive data on important procedural confounders such as contrast volume and intra-procedural hemodynamic instability. The inability to adjust for these factors in our multivariable model represents a significant limitation, as these are well-established contributors to the pathogenesis of PCI-associated AKI. Future studies should be designed to prospectively collect these data to allow for a more robust adjustment. Of note, a key limitation is reliance on a single 48-hour RNA sampling timepoint. Serial measurements across multiple timepoints (6, 12, 24, and 48 h post-PCI) would better characterize biomarker kinetics and refine predictive performance, particularly for distinguishing transient from persistent AKI. We also lacked long-term follow-up data and, critically, pre-PCI baseline measurements for *GTF2I*, *ANGPTL4*, and *MMP14*. Additionally, RNA biomarkers were assessed only at 48 h post-PCI, preventing characterization of temporal expression dynamics. Future studies should incorporate serial measurements to distinguish pre-existing from PCI-induced expression patterns.

From a clinical implementation perspective, although qPCR-based RNA assays are available in many tertiary centers, translation into routine practice requires several prerequisites. These include standardization of RNA extraction and analytical protocols, validation of reproducible clinical cut-off values across diverse populations, assessment of cost-effectiveness, and demonstration that biomarker-guided early detection translates into improved clinical outcomes. In addition, the development of rapid, automated testing platforms with shorter turnaround times would be essential to ensure real-world applicability in acute care settings.

Finally, validation in larger, more diverse populations with varying cardiovascular and renal disease severity is required to establish these biomarkers for routine clinical application.

## Conclusions

This study demonstrates that the molecular biomarkers *GTF2I*, *ANGPTL4*, and *MMP14* provide superior diagnostic accuracy compared with conventional markers for the early detection of AKI in patients with ACS undergoing PCI. Notably, the combined biomarker panel exhibited exceptional predictive performance (AUC = 0.99), underscoring its strong potential for clinical translation. From a clinical perspective, this panel may serve as an early risk-stratification tool to identify ACS patients at high risk of AKI following PCI, thereby enabling intensified renal monitoring and the timely implementation of preventive strategies. Importantly, measurement of this RNA biomarker panel at 48 h post-PCI could facilitate the identification of high-risk patients before elevations in conventional renal markers occur, supporting earlier nephroprotective interventions and potentially improving clinical outcomes.

## Supplementary Information

Below is the link to the electronic supplementary material.

Supplementary Material 1

Supplementary Material 2

## Data Availability

Data will be made available upon reasonable request.

## Abbreviations

ACS: Acute Coronary Syndrome
ADQI: Acute Disease Quality Initiative
AKD: Acute Kidney Disease
AKI: Acute Kidney Injury
ALT: Alanine Aminotransferase
AMI: Acute Myocardial Infarction
AST: Aspartate Aminotransferase
cANGPTL4: C-terminal Fibrinogen-Like Domain
ccRCC: Clear Cell Renal Cell Carcinoma
cDNA: Complementary DNA
CKD: Chronic Kidney Disease
CKD-EPI: Chronic Kidney Disease Epidemiology Collaboration
DEGs: Differentially Expressed Genes
ECG: Electrocardiographic
eGFR: Estimated Glomerular Filtration Rate
GEO: Gene Expression Omnibus
GTF2I: General Transcription Factor Ⅱi
HbA1c: Hemoglobin A1c
HDLc: High-Density Lipoprotein Cholesterol
IL-18: interleukin-18
KDIGO: Kidney Disease: Improving Global Outcomes
KIM-1: kidney injury molecule-1
LDLc: Low-Density Lipoprotein Cholesterol
LN: Lupus Nephritis
LPLs: Lipoprotein Lipases
MCD: Minimal Change Disease
MI: Myocardial Infarction
MMPs: Matrix Metalloproteinases
NIDDK: National Institute of Diabetes and Digestive and Kidney
NGAL: Neutrophil gelatinase-associated lipocalin
NSTE-ACS: Non-ST-Segment Elevation Acute Coronary Syndrome
PCI: Percutaneous Coronary Intervention
SLE: Systemic Lupus Erythematosus
STEMI: ST-Segment Elevation Myocardial Infarction
TC: Total Cholesterol
TG: Triglycerides
UA: Unstable Angina
VE-Cad: Vascular-Endothelial Cadherin
VEGF: Vascular Endothelial Growth Factor
