# Supplementary material for: RNA biomarker signatures for prediction of acute kidney injury in acute coronary syndrome patients undergoing PCI
Source: BMC Nephrol. 2026 Apr 14;27:242. doi: 10.1186/s12882-026-04926-w (PMC13088838; doi:10.1186/s12882-026-04926-w)

**Table S2** Details of the GSE116626, GSE19339, and GSE36895datasets that were retrieved from the GEO database.

| Accession Number | Platform | Organism | Experiment type | Number of samples | Experimental design |
| --- | --- | --- | --- | --- | --- |
| GSE116626 | GPL14951 Illumina HumanHT-12 WG-DASL V4.0 R2 expression beadchip | *Homo sapiens* | Expression profiling by array | 52: chronic renal patients  29: Control | The study analyzed kidney biopsy samples from three groups: 52 IgA nephropathy (IgAN) patients, in addition to 22 non-IgA glomerulonephritis (non-IgAN GN), and 7 living kidney donors as control groups. RNA was extracted to generate gene expression profiles, focusing on biomarkers linked to active and chronic renal lesions. |
| GSE19339 | GPL570 [HG-U133_Plus_2] Affymetrix Human Genome U133 Plus 2.0 Array | Homo sapiens | Expression profiling by array | 4: MI  4: Control | Thrombi of ACS patients were harvested from the site of coronary occlusion. Leukocytes were isolated by Ficoll centrifugation. Peripheral blood leukocytes (PBL) were treated in a similar fashion and mRNA was extracted from both cells. |
| GSE36895 | GPL570 [HG-U133_Plus_2] Affymetrix Human Genome U133 Plus 2.0 Array | Homo sapiens | Expression profiling by array | 29: ccRCC  23: Control | The RNA of clear-cell renal cell carcinoma (ccRCC) primary tumors and normal kidney cortices were labeled and hybridized to Affymetrix Human Genome U133 Plus 2.0 arrays. |

**Table S3 Spearman’s Correlation Coefficients Between Biomarkers and Clinical Indicators**

| **Correlations** | | | | | | | | | |
| --- | --- | --- | --- | --- | --- | --- | --- | --- | --- |
|  | | | GTF2I | ANGPTL4 | MMP14 | Cystatin C | creatinine | CK-MB | Troponin |
| Spearman's rho | GTF2I | Correlation Coefficient | 1.000 | .489^**^ | .435^**^ | .280^**^ | .065 | .545^**^ | .547^**^ |
|  |  | Sig. (2-tailed) | . | .000 | .000 | .000 | .405 | .000 | .000 |
|  |  | N | 167 | 167 | 167 | 167 | 167 | 167 | 167 |
|  | ANGPTL4 | Correlation Coefficient | .489^**^ | 1.000 | .383^**^ | .323^**^ | -.213^**^ | .567^**^ | .492^**^ |
|  |  | Sig. (2-tailed) | .000 | . | .000 | .000 | .006 | .000 | .000 |
|  |  | N | 167 | 167 | 167 | 167 | 167 | 167 | 167 |
|  | MMP14 | Correlation Coefficient | .435^**^ | .383^**^ | 1.000 | .308^**^ | -.076 | .396^**^ | .432^**^ |
|  |  | Sig. (2-tailed) | .000 | .000 | . | .000 | .330 | .000 | .000 |
|  |  | N | 167 | 167 | 167 | 167 | 167 | 167 | 167 |
|  | Cystatin C | Correlation Coefficient | .280^**^ | .323^**^ | .308^**^ | 1.000 | -.203^**^ | .226^**^ | .373^**^ |
|  |  | Sig. (2-tailed) | .000 | .000 | .000 | . | .009 | .003 | .000 |
|  |  | N | 167 | 167 | 167 | 167 | 167 | 167 | 167 |
|  | Creatinine | Correlation Coefficient | .065 | -.213^**^ | -.076 | -.203^**^ | 1.000 | -.095 | -.028 |
|  |  | Sig. (2-tailed) | .405 | .006 | .330 | .009 | . | .223 | .723 |
|  |  | N | 167 | 167 | 167 | 167 | 167 | 167 | 167 |
|  | CK-MB | Correlation Coefficient | .545^**^ | .567^**^ | .396^**^ | .226^**^ | -.095 | 1.000 | .576^**^ |
|  |  | Sig. (2-tailed) | .000 | .000 | .000 | .003 | .223 | . | .000 |
|  |  | N | 167 | 167 | 167 | 167 | 167 | 167 | 167 |
|  | Troponin | Correlation Coefficient | .547^**^ | .492^**^ | .432^**^ | .373^**^ | -.028 | .576^**^ | 1.000 |
|  |  | Sig. (2-tailed) | .000 | .000 | .000 | .000 | .723 | .000 | . |
|  |  | N | 167 | 167 | 167 | 167 | 167 | 167 | 167 |
| **. Correlation is significant at the 0.01 level (2-tailed). | | | | | | | | | |

*CK-MB creatine kinase-MB.*

**Table S4** Univariate screening of clinical and molecular variables associated with AKI.

| **Variables in the Equation** | | | | | | | |
| --- | --- | --- | --- | --- | --- | --- | --- |
|  | | **B** | **S.E.** | **Wald** | **Df** | **Sig.** | **Exp(B)** |
| Step 0 | Constant | .636 | .238 | 7.141 | 1 | .008 | 1.889 |

| Variables not in the Equation^a^ | | | | | |
| --- | --- | --- | --- | --- | --- |
|  | | | Score | Df | Sig. |
| Step 0 | Variables | Normoalbuminuric | 78.000 | 2 | .000 |
|  |  | Microalbuminuric | 78.000 | 1 | .000 |
|  |  | Macroalbuminuric | 20.647 | 1 | .000 |
|  |  | ANGPTL14 | 31.685 | 1 | .000 |
|  |  | GTF2I | 4.673 | 1 | .031 |
|  |  | MMP14 | 4.899 | 1 | .027 |
|  |  | Cystatin C | 1.093 | 1 | .296 |
|  |  | Age | .466 | 1 | .495 |
|  |  | Sex | .459 | 1 | .498 |
|  |  | Non-smoker | 2.907 | 2 | .234 |
|  |  | Smoker | 2.802 | 1 | .094 |
|  |  | X-Smoker | 1.483 | 1 | .223 |
|  |  | Family History | .147 | 1 | .701 |
|  |  | Not Diabetic | 18.519 | 3 | .000 |
|  |  | <5 years diabetic | 1.913 | 1 | .167 |
|  |  | 5-10 years diabetic | 12.278 | 1 | .000 |
|  |  | >10 years diabetic | .866 | 1 | .352 |
|  |  | Fasting Glucose | .039 | 1 | .843 |
|  |  | Post Prandial Glucose | 2.866 | 1 | .090 |
|  |  | HbA1c | 19.577 | 1 | .000 |
|  |  | Insulin | .844 | 1 | .358 |
|  |  | HOMA-IR | .025 | 1 | .875 |
|  |  | HOMA-B | 14.912 | 1 | .000 |
|  |  | Systolic blood pressure (mmHg) | .00016 | 1 | .990 |
|  |  | Diastolic blood pressure (mmHg) | .264 | 1 | .607 |
|  |  | BMI | .369 | 1 | .544 |
|  |  | Total Cholesterol | .520 | 1 | .471 |
|  |  | LDL | .290 | 1 | .590 |
|  |  | HDL | 2.718 | 1 | .099 |
|  |  | TGs | 7.165 | 1 | .007 |
|  |  | Serum Creatinine | 3.999 | 1 | .046 |
|  |  | eGFR | 47.284 | 1 | .000 |
|  |  | Alb/creat ratio | 1.533 | 1 | .216 |
|  |  | ALT | 1.133 | 1 | .287 |
|  |  | AST | .079 | 1 | .779 |
|  |  | CKMB | .184 | 1 | .668 |
|  |  | Troponin | 2.767 | 1 | .096 |
| a. Residual Chi-Squares are not computed because of redundancies. | | | | | |

*ACS acute coronary syndrome, AKI acute kidney injury, Alb/creat ratio albumin-to-creatinine ratio, ALT alanine aminotransferase, AST aspartate aminotransferase, BMI body mass index, CK-MB creatine kinase-MB, ECG electrocardiography, eGFR estimated glomerular filtration rate, HbA1c glycated hemoglobin A1c, HDL high-density lipoprotein, HOMA-B homeostatic model assessment of β-cell function, HOMA-IR homeostatic model assessment of insulin resistance, LDL low-density lipoprotein, PCI percutaneous coronary intervention, TGs triglycerides, Troponin cardiac troponin I.*

**Table S5. Multivariable logistic regression analysis identifying independent predictors of AKI.**

| **Variables in the Equation** | | | | | | | |
| --- | --- | --- | --- | --- | --- | --- | --- |
|  | | B | S.E. | Wald | df | Sig. | Exp(B) |
| Step 0 | Constant | -.094 | .194 | .233 | 1 | .629 | .911 |

| **Variable** | **Score Statistic** | **df** | ***P*-value** | **OR (95% CI)** | **Interpretation** |
| --- | --- | --- | --- | --- | --- |
| **Albuminuria (Overall)** | 107.000 | 2 | <0.001 | 4.50 (3.20–6.33) | Highly significant predictor (p <0.001) |
| **Albuminuria Category 1** | 107.000 | 1 | <0.001 | 5.20 (3.85–7.02) | Strong association with AKI risk |
| **Albuminuria Category 2** | 37.713 | 1 | <0.001 | 3.75 (2.40–5.85) | Significant association with AKI risk |
| ***ANGPTL14*** | 56.618 | 1 | <0.001 | 0.35 (0.25–0.50) | Very strong predictor (protective effect)* |
| ***GTF2I*** | 8.309 | 1 | 0.004 | 1.80 (1.20–2.70) | Significant predictor (p = 0.004) |
| ***MMP14*** | 10.172 | 1 | 0.001 | 2.10 (1.45–3.05) | Highly significant predictor (p = 0.001) |
| **Diabetes (Yes)** | 37.966 | 1 | <0.001 | 3.90 (2.60–5.85) | Major risk factor for AKI (p <0.001) |
| **Overall Model** | 107.000 | 6 | <0.001 | – | Combined predictors strongly improve model fit |

Abbreviations: ACS, acute coronary syndrome; AKI acute kidney injury.

**Fig S1** The protein-protein interaction of the selected mRNAs using the GeneMANIA database (<https://genemania.org/>, accessed January 2024)
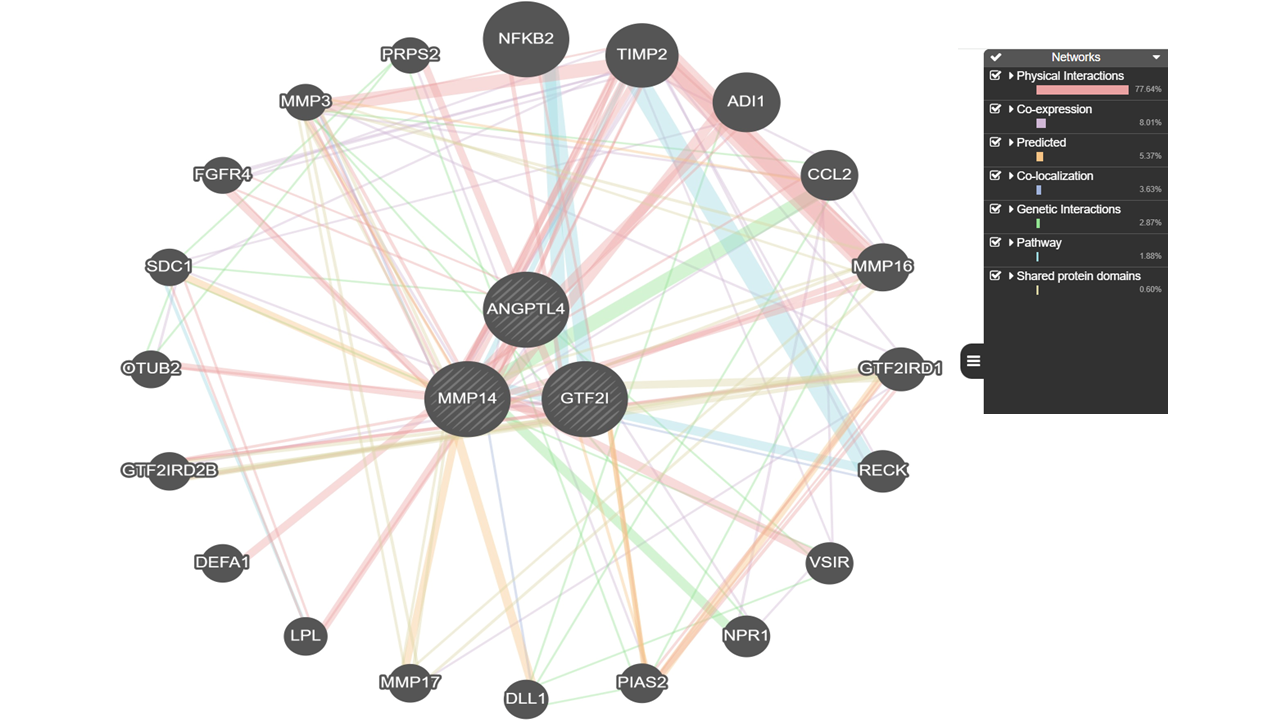


**
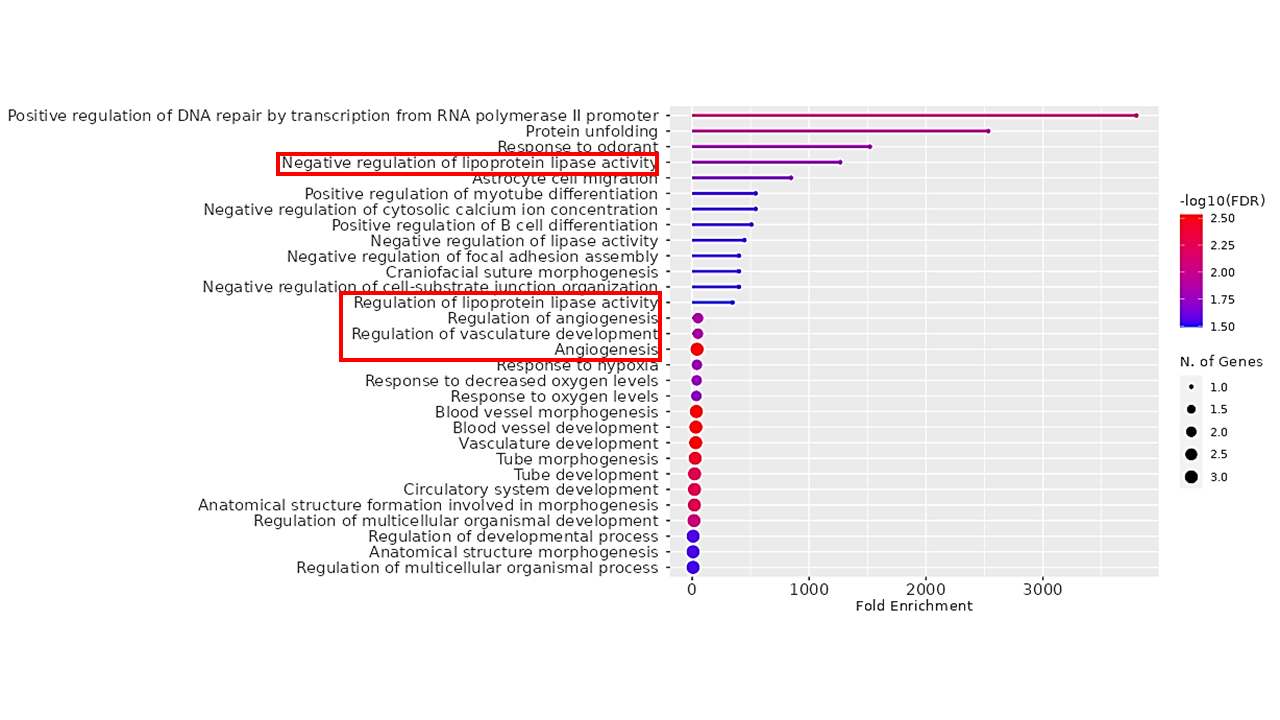
Fig S2** Gene Ontology analysis of *GTF2I, ANGPTL4*, and *MMP14* using the ShinyGO 0.80 (<http://bioinformatics.sdstate.edu/go/>, accessed January 2024).

**Fig S3** Gene Ontology analysis of *GTF2I, ANGPTL4*, and *MMP14* using GeneCards databases (<https://www.genecards.org/>, accessed January 2024).


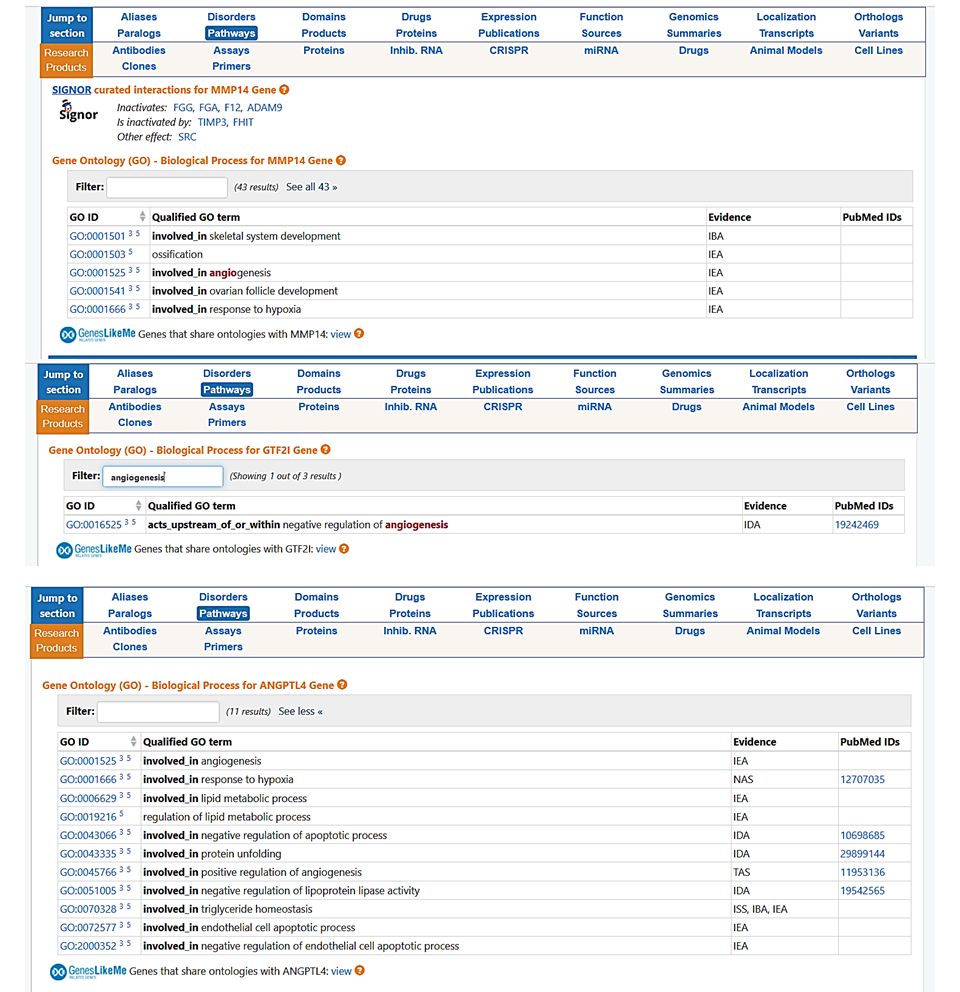


**Fig S4 Differential Expression of Serum *GTF2I*, *ANGPTL4*, and *MMP14* Across the Studied Groups. (a) the relative quantification of *GTF2I*, (b) the relative quantification of *ANGPTL4*, and (c) the relative quantification of *MMP14*.**


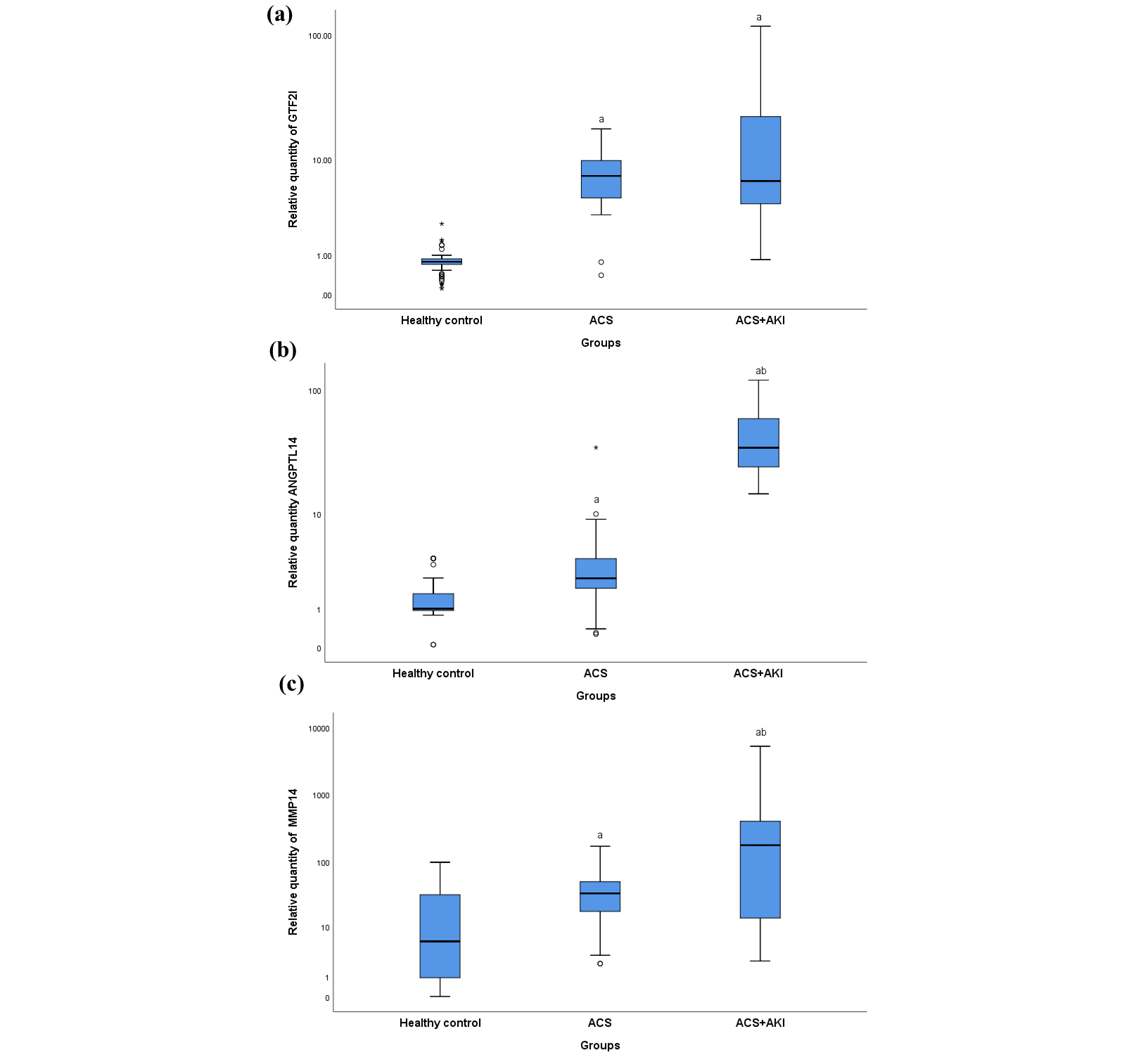


*ACS acute coronary syndrome, AKI acute kidney injury*

**Fig S5** ROC Curve Analysis for Biomarkers in Differentiating Healthy Controls from ACS Patients. (a) *GTF2I*, (b) *ANGPTL4*, (c) *MMP14*, (d) Combined (*GTF2I, ANGPTL4,* and *MMP14*), (e) Cystatin C, (f) Creatinine.


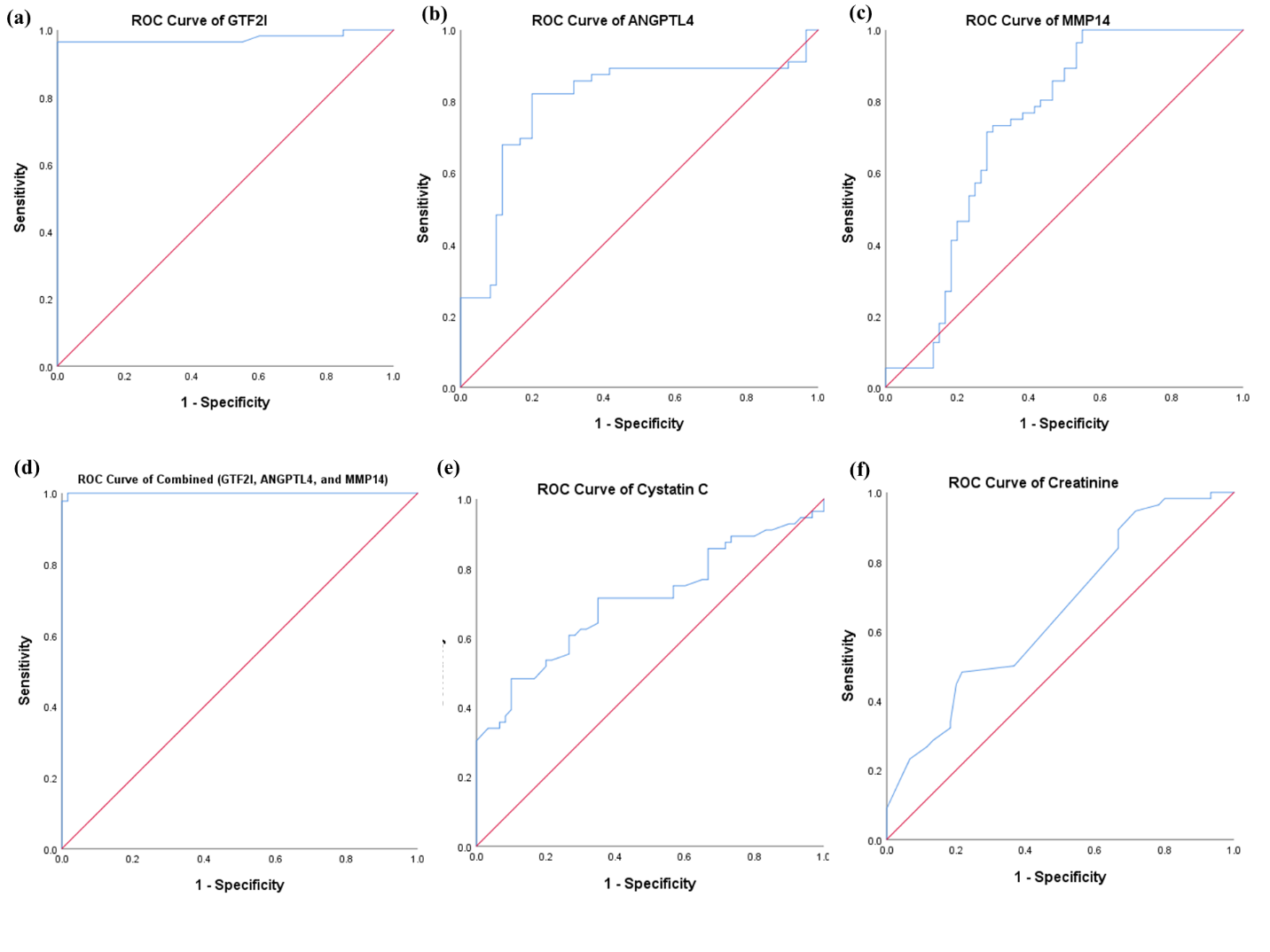


**Fig S6** ROC Curve Analysis for Biomarkers in Differentiating Healthy Controls from AACS-KI Patients. (a) *GTF2I*, (b) *ANGPTL4*, (c) *MMP14*, (d) Combined (*GTF2I, ANGPTL4,* and *MMP14*), (e) Cystatin C, (f) Creatinine.


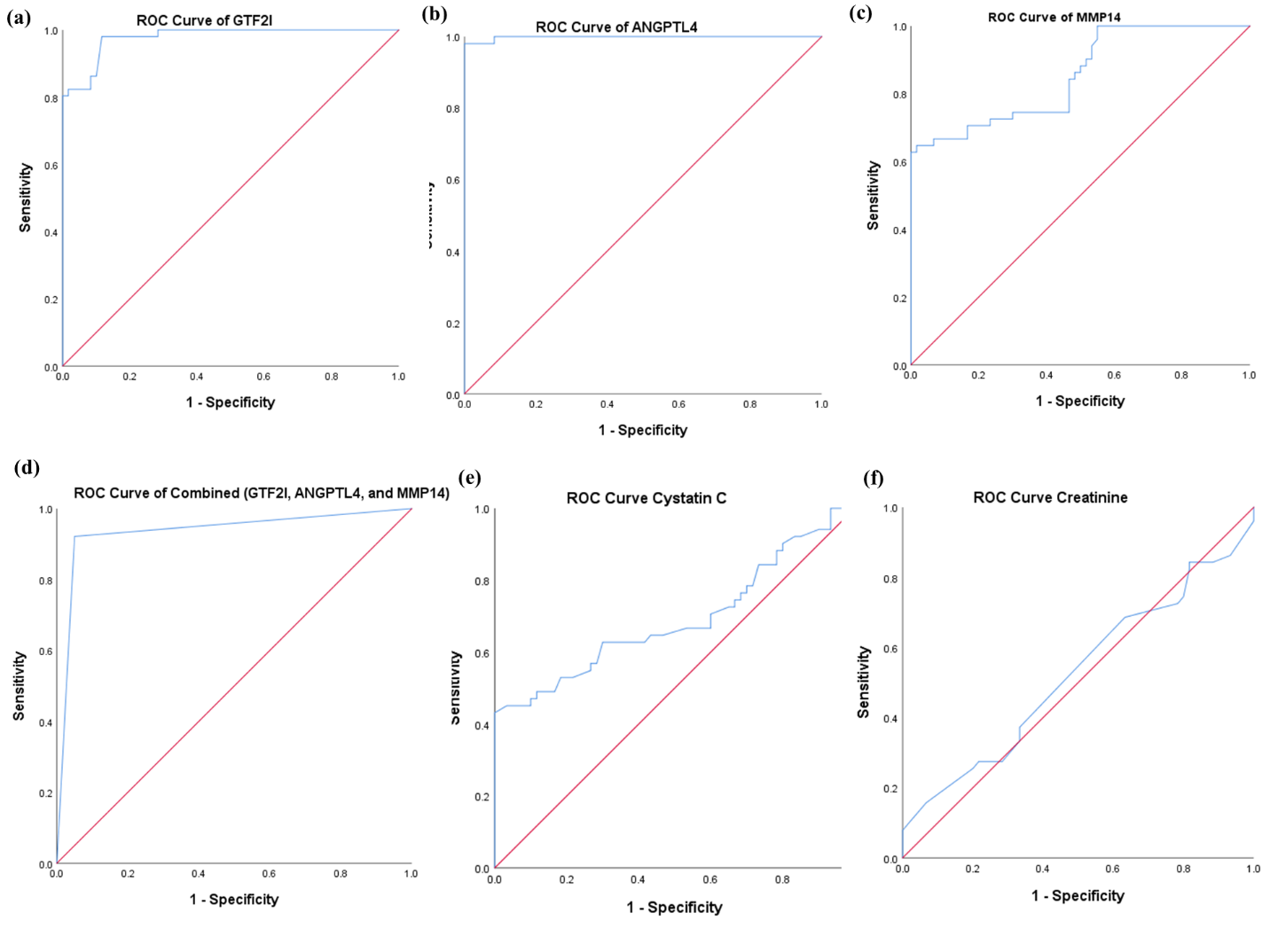


**
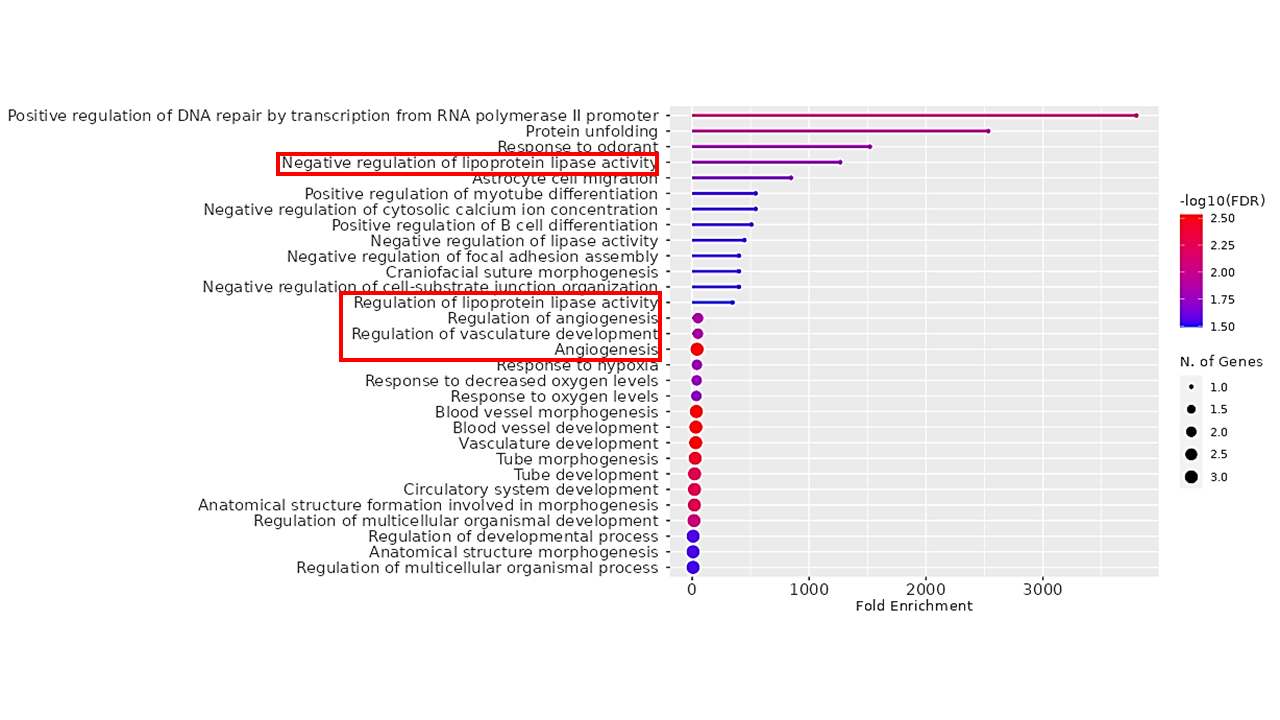
**

**Fig S7** ROC Curve Analysis for Biomarkers in Differentiating ACS from ACS-AKI Patients. (a) *GTF2I*, (b) *ANGPTL4*, (c) *MMP14*, (d) Combined (*GTF2I, ANGPTL4,* and *MMP14*), (e) Cystatin C, (f) Creatinine.


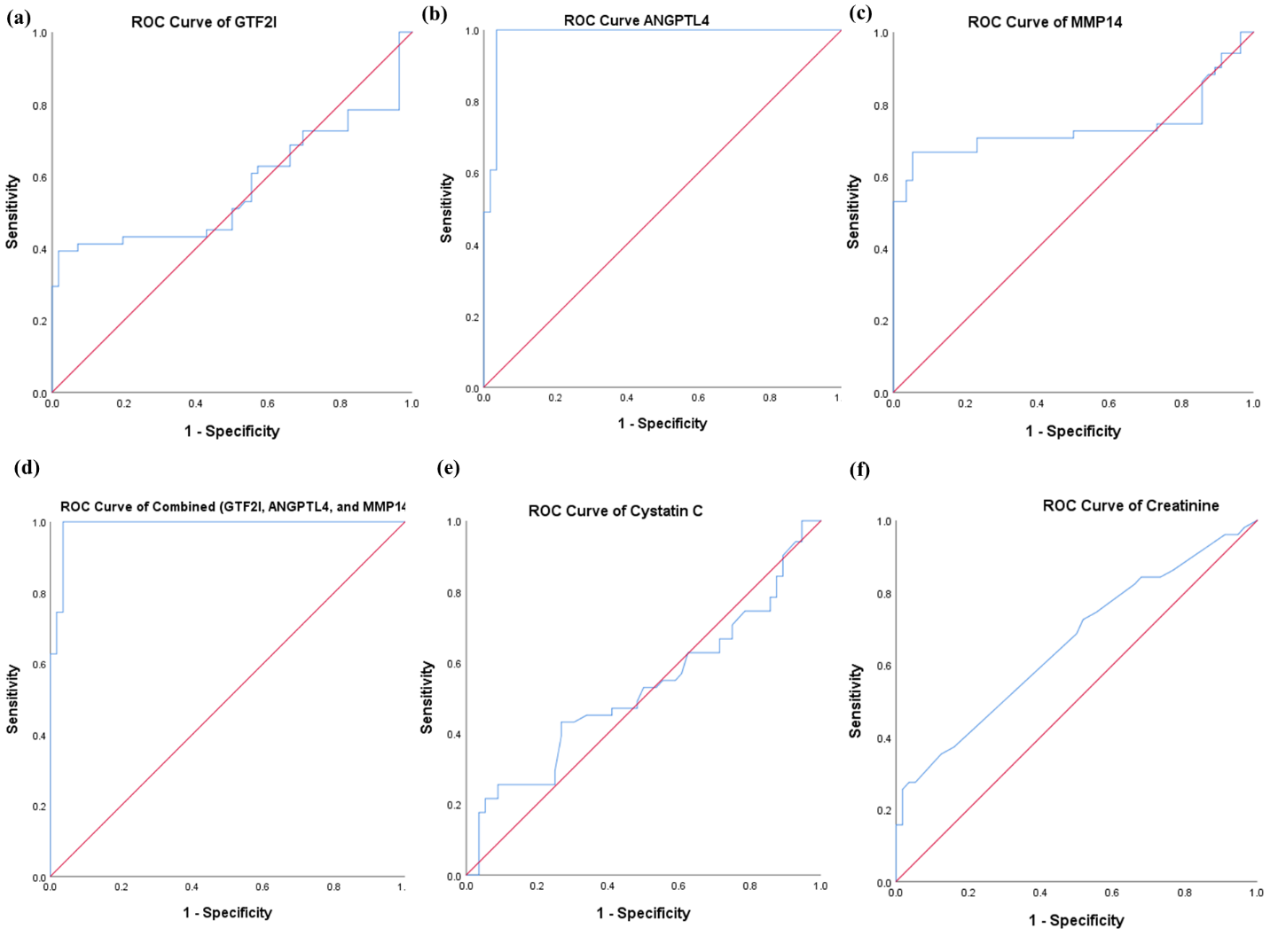

Supplement: Supplementary file 2 — Supplementary Material 2 [file 12882_2026_4926_MOESM2_ESM.docx]
